# A Hand-Book of Skin Diseases

**Published:** 1884-01

**Authors:** 


### BOOK NOTICES.

A Hand-book of Skin Diseases and their Homœopathic Treatment. By John R. Kippax, M. D., LL. B., etc. Second edition. Pp. 288. Chicago: Duncan Brothers, 1884.

To the busy practitioner, as well to the student, who desires to quickly gather a hint as to the diagnosis or treatment of any skin affection, Dr. Kippax's hand-book will be of service. The local treatment he recommends is often of positive injury, and seldom accomplishes lasting benefit. In treating these "skin diseases," the totality of the symptoms —internal as well as mental—must be carefully considered.

